# Correlation of clinical outcomes with bremsstrahlung and Y-90 PET/CT imaging findings following Y-90 radiosynoviorthesis: a prospective study

**DOI:** 10.1186/s13550-016-0201-z

**Published:** 2016-05-31

**Authors:** Thomas W. Barber, Martin H. Cherk, Anne Powell, Kenneth S. K. Yap, Baki Billah, Victor Kalff

**Affiliations:** Department of Nuclear Medicine & PET, The Alfred Hospital, Melbourne, 3004 Australia; Department of Medicine, Monash University, Alfred Hospital Campus, Melbourne, 3004 Australia; Department of Rheumatology, The Alfred Hospital, Melbourne, 3004 Australia; Department of Epidemiology and Preventive Medicine, Monash University, Alfred Hospital Campus, Melbourne, 3004 Australia

**Keywords:** Radiosynoviorthesis, Radiation synovectomy, Yttrium, PET/CT, SPECT/CT, Bremsstrahlung

## Abstract

**Background:**

It is unclear how to predict which patients will respond to Y-90 radiosynoviorthesis. The aim of this study is to correlate clinical outcomes following Y-90 radiosynoviorthesis with bremsstrahlung and Y-90 PET/CT imaging findings.

**Methods:**

Fifty-one joints underwent bremsstrahlung planar and Y-90 PET/CT imaging following Y-90 radiosynoviorthesis. The Y-90 distribution pattern on bremsstrahlung planar imaging was classified as diffuse or non-diffuse and compared with the intra or extra-articular location of activity on Y-90 PET/CT. Treatment response was assessed by patients and clinicians at 6 months. In patients who underwent bremsstrahlung SPECT, side-by-side comparison with PET was performed with image quality/resolution scored using a five-point-scale.

**Findings:**

Bremsstrahlung planar images were classified as diffuse in 33/51 (65 %) and non-diffuse in 18/51 (35 %) scans. There was no association between treatment response and the bremsstrahlung planar imaging pattern. PET/CT confirmed an intra-articular location in all 33/33 (100 %) diffuse scans and an extra-articular location in 3/18 (17 %) non-diffuse scans. Of the three joints with extra-articular activity, none had any treatment response. Excluding these three joints, there remained no association between the bremsstrahlung planar imaging pattern and treatment response. Of the 42 joints imaged with SPECT, PET image quality/resolution was classified as superior in 40 (95 %). In one patient with extra-articular activity on PET/CT, SPECT/CT was unable to definitively localise the activity to the intra or extra-articular space.

**Conclusions:**

The distribution pattern on bremsstrahlung planar imaging did not correlate with clinical outcome following Y-90 radiosynoviorthesis in our study population. However, in patients with non-diffuse planar imaging patterns, Y-90 PET/CT should be considered to exclude extra-articular activity with PET providing superior image quality compared to SPECT.

## Findings

### Introduction

Radiosynoviorthesis using Y-90 is a treatment option for refractory synovitis. Despite being used for over 50 years, the reported efficacy is variable and it remains unclear how to predict which patients will respond to treatment [1–5].

The hypothesised mechanism of action involves phagocytosis of Y-90 by synovial cells resulting in local radiation induced necrosis from the emitted β-rays [6]. Imaging the distribution of Y-90 may be achieved via the associated bremsstrahlung radiation resulting from the deceleration of β-rays in tissues. Two dimensional planar images are typically performed following treatment, although definitive localization to the intra- or extra-articular space is not possible with this technique and the reasons for non-diffuse planar distribution patterns remain unclear [2, 7]. More precise anatomical localization may be obtained using three-dimensional bremsstrahlung single-photon emission computed tomography (SPECT) fused with X-ray computed tomography (CT); however, this technique does not overcome the limited spatial resolution inherent with bremsstrahlung imaging. More recently, it has been recognised that a minor amount of positrons are emitted in the Y-90 decay scheme making imaging with position emission tomography (PET) possible [8–10]. Y-90 PET imaging has been reported to result in superior image quality/resolution compared to SPECT and may provide a more accurate assessment of the intra- or extra-articular distribution of Y-90 [11]. Given that patients with extra-articular activity are unlikely to respond to treatment, Y-90 PET/CT has the potential to aid in predicting treatment response and also identify patients at risk for local complications. This is particularly relevant given that current post-treatment planar and SPECT imaging techniques have an uncertain role in the prediction of clinical effect [2, 7].

Therefore, the aim of this study is to compare planar bremsstrahlung imaging patterns with the intra- or extra-articular location of activity on Y-90 PET/CT and to correlate imaging findings with clinical outcomes following radiosynoviorthesis.

### Materials and methods

#### Patients

Fifty-one joints (32 knees, 17 ankles and 2 elbows) in 41 patients were treated with Y-90 radiosynoviorthesis. All patients were treated for refractory synovitis caused by haemophilia (25 joints), seronegative arthritis (19 joints), rheumatoid arthritis (4 joints) or other causes of arthropathy (3 joints). All patients provided informed consent with the study approved by our institutional hospital ethics committee.

#### Y-90 radiosynoviorthesis procedure

Radiosynoviorthesis of the knee was performed using 185–222 MBq of Y-90 citrate colloid which was administered following the confirmation of intra-articular positioning by either aspiration of synovial fluid (21 knees) or by using fluoroscopic guidance (11 knees). Radiation synovectomy of the ankles and elbows was performed using 74 MBq of Y-90 citrate colloid under fluoroscopic guidance. Our institutional protocol also includes intra-articular coadministration of the corticosteroid Depomedrol (80 mg for knees, 40 mg for ankles and 20 mg for elbows). Patients were admitted to hospital following the procedure with treated joints splinted for 48 h to ensure immobilisation.

#### Imaging

Imaging was performed on all patients between 4 and 24 h following Y-90 administration. Bremsstrahlung planar imaging was performed on all joints with images acquired for 2 min with a broad energy window (up to 70–210 keV) using medium energy general purpose collimators. Bremsstrahlung planar imaging of the draining nodal stations and liver/spleen was also performed on all patients to assess for nodal and systemic leakage, respectively. PET/CT imaging was performed on all joints using a Philips GEMINI PET/CT scanner (Philips Medical Systems, Cleveland, OH, USA) with an acquisition time of between 15 and 20 min. PET images were reconstructed using 3D row action maximum likelihood algorithm iterative reconstruction using two iterations. Bremsstrahlung SPECT or SPECT/CT was performed on 42 joints using either a General Electric Discovery VH (Milwaukee, WI, USA) gamma camera with a 1-in.-thick crystal, a General Electric Discovery 670 (Milwaukee, WI, USA) gamma camera with a 5/8-in.-thick crystal or a Siemens e.cam (Hoffman Estates, IL, USA) gamma camera with a 1-in.-thick crystal. SPECT images were acquired with a broad energy window (up to 70–210 keV) using medium energy general purpose collimators and a 128 × 128 matrix with 60–64 projections (25–36 s per step). SPECT data were processed using GE’s Volumetrix MI program on a Xeleris 3 workstation (Milwaukee, WI, USA) incorporating iterative reconstruction and resolution recovery software. Attenuation correction was also incorporated for joints imaged with SPECT/CT.

#### Image assessment

The distribution pattern of Y-90 on planar bremsstrahlung imaging was scored based on the criteria outlined by Jahangier et al. [1] and classified as either (I) diffuse, (II) predominantly diffuse but also focal, (III) predominantly focal but also diffuse or (IV) focal. These classifications were divided into two groups: diffuse (class I) and non-diffuse (classes II, III and IV) for statistical analysis. The intra- or extra-articular location of Y-90 was assessed on fused PET/CT. In patients who underwent SPECT, side-by-side comparison with PET was performed and image quality/resolution scored using a 5-point scale according to the following criteria: 5 = PET definitely superior; 4 = PET probably superior; 3 = PET equivalent; 2 = PET probably inferior and 1 = PET definitely inferior.

#### Clinical assessment

Clinical outcome involved the assessment of seven variables both before and 6 months after treatment by patients and treating rheumatologists (43 joints assessed prospectively and 8 joints assessed retrospectively). Each variable was assigned a score based on the composite change index (CCI) criteria outlined by Jahangier et al. [1]. Details of the seven variables assessed were as follows. (1) Pain: assessed using a visual analogue scale (VAS; 0 = no pain; 100 = maximal pain). For 30–50 mm improvement, 1 point was added to the CCI; for a positive change >50 mm, 2 points were added. (2) Functional disability score: assessed using a scale of 1–5 (1 = complete immobilisation, 2 = severe disability, 3 = moderate disability, 4 = slight disability, 5 = no disability). For a positive change of 1 disability level, 1 point was added to the CCI; for a positive change of ≥ 2 levels, 2 points were added. (3) Joint tenderness (0 = no tenderness, 1 = pain on pressure, 2 = pain and wincing on pressure, 3 = pain, wincing and withdrawal on pressure). For an improvement of 1 tenderness level, 1 point was added to the CCI; for improvement of ≥ 2 levels, 2 points were added. (4) Joint effusion (presence, absence). For resolution, 1 point was added to the CCI. (5) Joint swelling (presence, absence). For resolution, 1 point was added to the CCI. (6) Patient global response assessment (1 = no improvement or worsening of arthritis, 2 = mild improvement, 3 = moderate improvement, 4 = strong improvement). For mild, moderate and strong improvement, 0, 1 and 2 points, respectively, were added to the CCI. (7) Treating clinician global response assessment (1 = no improvement or worsening of arthritis, 2 = mild improvement, 3 = moderate improvement, 4 = good improvement, 5 = excellent improvement, 6 = resolution of arthritis). For mild, moderate and good (or greater) improvement, 0, 1 and 2 points, respectively, were added to the CCI. A successful overall response was defined as CCI ≥ 6.

#### Statistical analysis

Comparison of patient demographics, clinical response variables and extra-articular activity between the groups with diffuse and non-diffuse planar imaging Y-90 distributions was performed using the chi-square test, Fisher’s exact test, Wilcoxon-Mann-Whitney test and unpaired Student’s *t* test. Results have been reported as frequency (%), mean +/− standard deviation and median with interquartile range. A two-sided *p* value <0.05 was considered statistically significant. Analysis was performed using Analyse-it for Microsoft Excel version 2.20 (Analyse-it Software, Ltd. http://www.analyse-it.com/; 2009).

### Results

#### Patient characteristics

Fifty-one joints in 41 patients were treated with Y-90 radiosynoviorthesis. Patient demographics and clinical characteristics are listed in Table 1. All patients were treated for refractory synovitis caused by haemophilia (25 joints) or inflammatory arthritis (26 joints). All joints were imaged with bremsstrahlung planar imaging and Y-90 PET/CT. Forty-two joints were also imaged with SPECT including 34 imaged with SPECT/CT. Clinical follow-up was available in all patients.

**Table 1:** Patient characteristics, treatment response and imaging findings

	Planar imaging pattern		*P* value
	Diffuse (*n* = 33)	Non-diffuse (*n* = 18)	
Age (mean +/− SD)	43 +/− 16	49 +/− 11	0.16
Sex			
M	22 (67 %)	10 (56 %)	0.43
F	11 (33 %)	8 (44 %)	
Indication			
Haemophilia	19 (58 %)	6 (33 %)	0.10
Non-haemophilia	14 (42 %)	12 (67 %)	
Clinical response			
Overall response (CCI ≥ 6)	8 (24 %)	6 (33 %)	0.49
Median CCI	2 (0.9–7.1)	4 (1.0–5.3)	0.40
CCI classification			
0–4	22 (67 %)	11 (61 %)	0.90
4–8	9 (27 %)	5 (28 %)	
8–12	2 (6 %)	2 (11 %)	
Improvement in clinical variables			
VAS	3 (9 %)	4 (22 %)	0.23
Disability	20 (61 %)	11 (61 %)	0.97
Tenderness	15 (45 %)	11 (61 %)	0.29
Effusion (*n* = 45)	14 (50 %)	6 (35 %)	0.34
Swelling (*n* = 47)	6 (21 %)	4 (22 %)	1.00
Patient assessment	10 (30 %)	8 (44 %)	0.31
Clinician assessment	14 (42 %)	10 (56 %)	0.37
Extra-articular Y-90 activity	0 (0 %)	3 (17 %)	0.04
Nodal leak	0 (0 %)	2 (11 %)	0.12
Systemic leak	0 (0 %)	0 (0 %)	1.00

*CCI* composite change index, *VAS* visual analogue scale

#### Clinical outcome and imaging findings

**Fig. 1:**
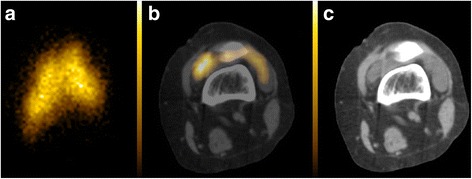
Anterior bremsstrahlung planar (**a**), axial fused PET/CT (**b**) and co-registered CT (**c**) images of the left knee demonstrating a diffuse planar imaging Y-90 pattern confirmed to be located intra-articularly on fused PET/CT

**Fig. 2:**
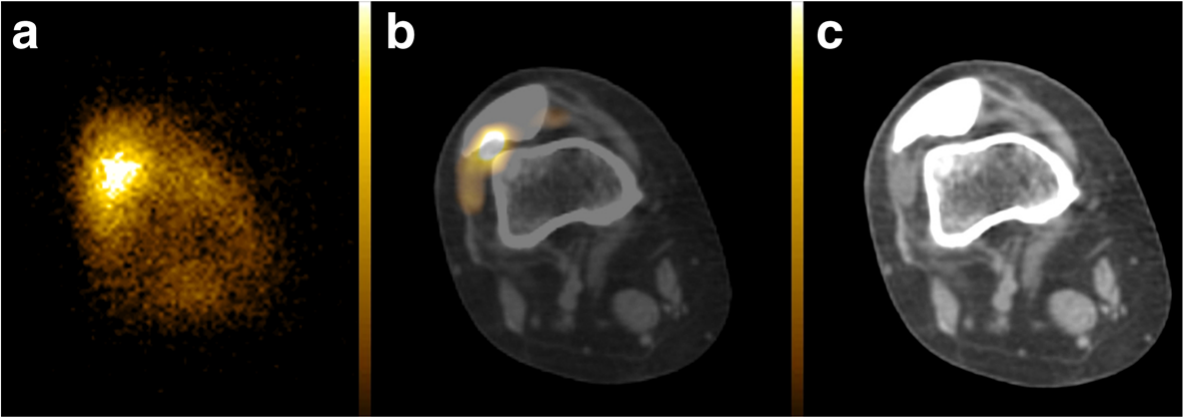
Anterior bremsstrahlung planar (**a**), axial fused PET/CT (**b**) and co-registered CT (**c**) images of the right knee demonstrating a predominantly diffuse but also focal (non-diffuse) planar imaging Y-90 pattern located intra-articularly on fused PET/CT

**Fig. 3:**
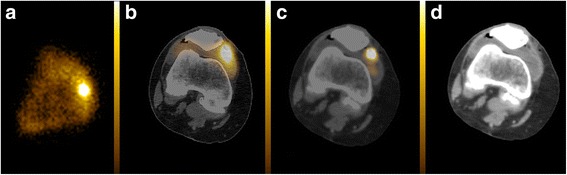
Anterior bremsstrahlung planar (**a**), axial fused SPECT/CT (**b**), axial fused PET/CT (**c**) and co-registered CT (**d**) images of the right knee demonstrating a predominantly diffuse but also focal (non-diffuse) planar imaging Y-90 pattern with the focal component of the Y-90 activity located extra-articularly on fused PET/CT (superficial to the deepest capsular layer but deep to the medial patellar retinaculum). SPECT/CT was unable to definitively localise the focal component to the intra- or extra-articular space

**Fig. 4:**
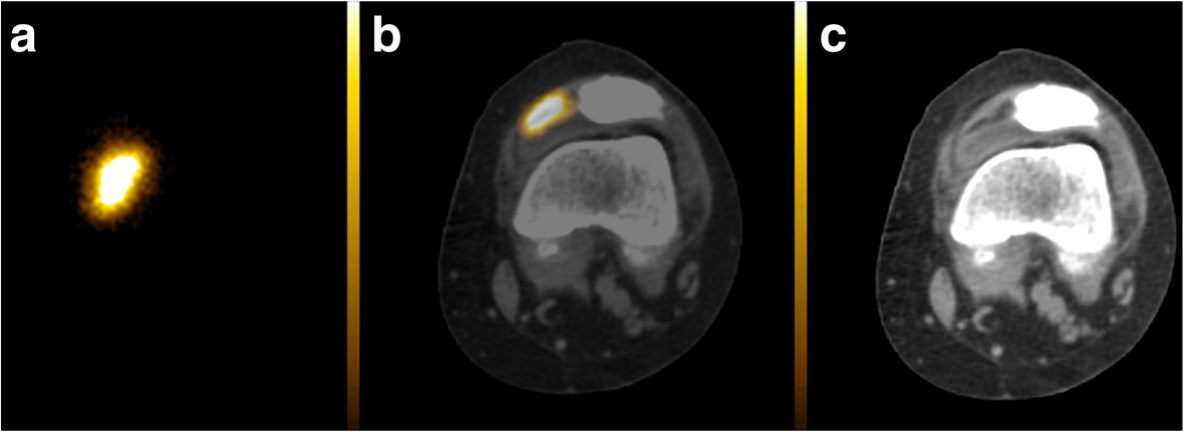
Anterior bremsstrahlung planar (**a**), axial fused PET/CT (**b**) and co-registered CT (**c**) images of the left knee demonstrating a focal (non-diffuse) planar imaging Y-90 pattern located extra-articularly on fused PET/CT (superficial to the deepest capsular layer but deep to the medial patellar retinaculum)

Bremsstrahlung planar images were classified as diffuse in 33/51 (65 %) and non-diffuse in 18/51 (35 %). Examples are shown in Figs. 1, 2, 3 and 4. Eight of the 33 joints with a diffuse pattern (24 %) and six of the 18 joints with a non-diffuse pattern (33 %) had an overall response to treatment (CCI ≥ 6). There was no association between the bremsstrahlung planar imaging pattern and overall treatment response (*p* = 0.49). There was no association between the bremsstrahlung planar imaging pattern and any of the treatment response variables (Table 1). In all 33 diffuse scans, PET/CT confirmed an intra-articular Y-90 distribution. Of the 18 non-diffuse scans, PET/CT demonstrated extra-articular Y-90 activity in three joints (17 %). Two of these patients had no detectable Y-90 activity within the joint space of the knee and one had both intra-articular and extra-articular Y-90 knee activity (Figs. 3 and 4). All three patients with extra-articular Y-90 activity on PET/CT imaging had intra-articular needle positioning confirmed during the procedure by aspiration of synovial fluid without fluoroscopic guidance. These three patients with extra-articular activity had no response to treatment (CCI = 0 in all) and no local complications at 6 months follow-up. Excluding these three patients, there remained no association between any of the treatment response variables and the bremsstrahlung planar imaging pattern.

Leakage to regional lymph nodes was demonstrated in two patients on 24 h imaging. Both these patients had a non-diffuse planar imaging pattern located intra-articularly on PET/CT. None of the three patients with extra-articular Y-90 activity had leakage to regional lymph nodes at 24 h. No patients had systemic leakage to the liver or spleen.

SPECT imaging was performed in 42 joints including 34 imaged with SPECT/CT. Of these 42 joints, PET image quality/resolution was scored as definitely superior to SPECT in 36, probably superior to SPECT in four, equivalent to SPECT in one and inferior to SPECT in one. In the one patient with both intra- and extra-articular knee joint activity on PET/CT, SPECT/CT had insufficient resolution to definitively localise the Y-90 activity to the intra- or extra-articular space (Fig. 3).

### Discussion

Predicting patient response to Y-90 radiosynoviorthesis remains problematic despite over 50 years of clinical experience. While a diffuse intra-articular Y-90 distribution pattern on imaging may be expected to provide a more widespread radiation dose, our results do not suggest this correlates with clinical outcome. Our results also do not definitively support the postulate that focal imaging patterns may predict improved clinical outcomes by reflecting Y-90 localisation to areas of synovial disease activity [12, 13]. There remained no association between the planar imaging pattern and clinical outcome even after exclusion of patients with extra-articular activity on Y-90 PET/CT. This is in keeping with the findings of Jahangier et al., although our study has extended upon these results by using Y-90 PET/CT to confirm an intra-articular location rather than relying on planar imaging alone [2].

Three patients in our study had extra-articular Y-90 activity, and none of these patients had any response to treatment. While all three had a non-diffuse pattern on planar imaging, the majority of patients with non-diffuse planar imaging patterns did not have extra-articular activity. Therefore, three-dimensional imaging (SPECT or PET) fused with CT is required for accurate localisation of Y-90 activity when there are non-diffuse patterns on planar imaging. While our study did not assess the cause of non-diffuse intra-articular Y-90 patterns, it has been suggested that intra-articular septae or large synovial folds could potentially hamper the Y-90 distribution [2]. The reasons for extra-articular Y-90 activity in the three patients in our study also remain unclear, although altered needle/joint capsule positioning after aspiration of synovial fluid is likely given that all three patients with extra-articular Y-90 activity had the procedure performed on the knee without fluoroscopic guidance.

Our results demonstrate that PET provides superior image quality/resolution compared to SPECT and PET/CT is therefore the preferred imaging modality in keeping with prior results from our institution [11]. This is important given that patients with extra-articular activity are unlikely to respond to treatment and are at risk for potentially serious local complications. Identification of extra-articular activity on Y-90 PET/CT may therefore expedite alternative therapeutic approaches and guide monitoring for potential sites of radionecrosis. On the other hand, diffuse planar imaging patterns were confirmed to be intra-articular in all patients in our series and therefore do not warrant SPECT/CT or PET/CT for localisation. Leakage of Y-90 to regional lymph nodes was demonstrated in only two patients in our series. These patients had non-diffuse planar imaging findings and an intra-articular location on PET/CT. None of the three patients with extra-articular Y-90 activity had evidence of nodal or systemic leak. These results suggest that leakage on imaging does not necessarily correlate with extra-articular activity.

Limitations of our study include the relatively small and heterogeneous patient population which may limit generalisation of our results. In particular, our patient cohort included a large proportion of patients with haemophilia-related synovitis while other studies assessing radiopharmaceutical distribution have focused on non-haemophilic inflammatory arthropathies [2, 7, 14]. Only performing imaging at a single time point (4 or 24 h) is another potential limitation, although recent evidence suggests that the Y-90 activity distribution on SPECT/CT imaging does not significantly alter over a 72 h period [14].

### Conclusion

The distribution pattern on bremsstrahlung planar imaging did not correlate with clinical outcome following Y-90 radiosynoviorthesis in our study population. However, in patients with non-diffuse planar imaging patterns, Y-90 PET/CT should be considered to exclude extra-articular activity with PET providing superior image quality compared to SPECT.
